# Supplementary material for: Arginine impacts aggregation, biofilm formation, and antibiotic susceptibility in Enterococcus faecalis
Source: bioRxiv. 2024 May 30:2024.05.30.596650. Preprint. [Version 1] doi: 10.1101/2024.05.30.596650 (PMC11160706; doi:10.1101/2024.05.30.596650)
Supplement: Supplement 2 [file NIHPP2024.05.30.596650v1-supplement-2.pdf]

bioRxiv preprint doi: https://doi.org/10.1101/2024.05.30.596650; this version posted May 31, 2024. The copyright holder for this preprint (which was not certified by peer review) is the author/funder, who has granted bioRxiv a license to display the preprint in perpetuity. It is made available under aCC-BY-NC-ND 4.0 International license.

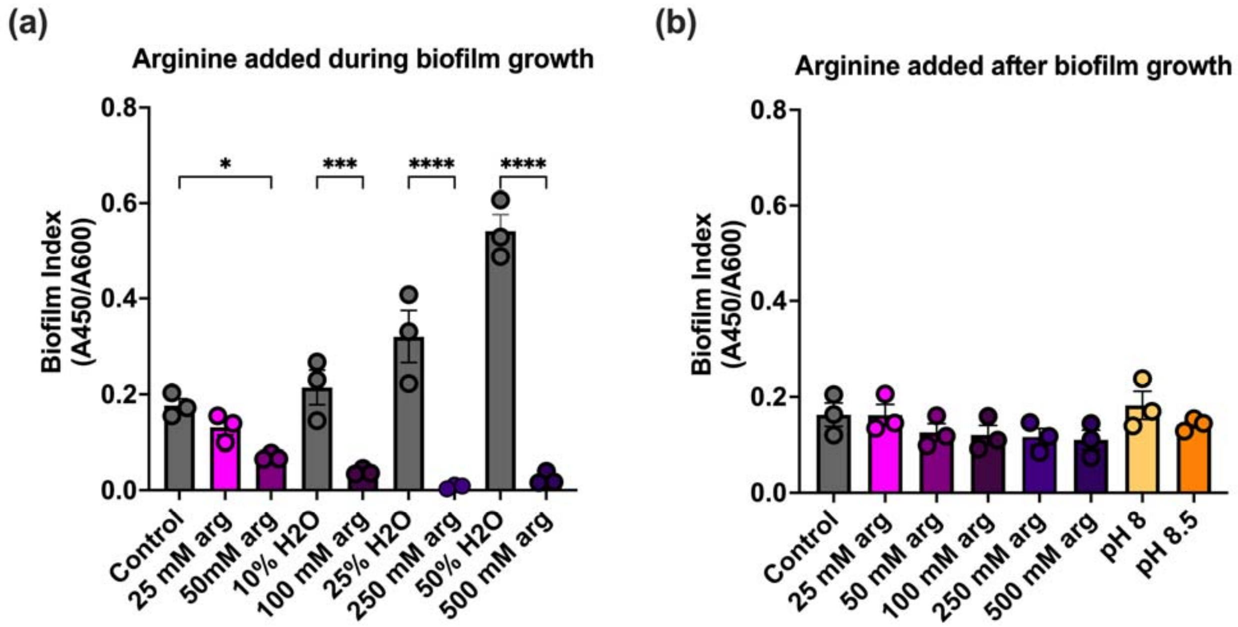

Supplementary Figure 1. *E. faecalis* biofilm formation in high concentrations of arginine. (a) *E. faecalis* OG1RF was grown for 24 hr with arginine added at the indicated concentrations. Due to the increasing volume of arginine in samples treated during growth, each arginine-treated sample is shown next to a volumetric control. **(b)** Biofilms were grown in the absence of arginine and washed, after which arginine, vehicle control, or Tris-HCl buffer at the indicated concentration were added for 20 min. For both panels, each data point represents an individual biological replicate. Statistical significance was evaluated using one-way ANOVA with Sidak's test (in panel **(a)**) or Dunnett's test (in panel **(b)**) for multiple comparisons. Comparisons that were not statistically significant are not shown. *$p<0.05$, **$p<0.01$, ***$p<0.001$, ****$p<0.0001$.

bioRxiv preprint doi: https://doi.org/10.1101/2024.05.30.596650; this version posted May 31, 2024. The copyright holder for this preprint (which was not certified by peer review) is the author/funder, who has granted bioRxiv a license to display the preprint in perpetuity. It is made available under aCC-BY-NC-ND 4.0 International license.

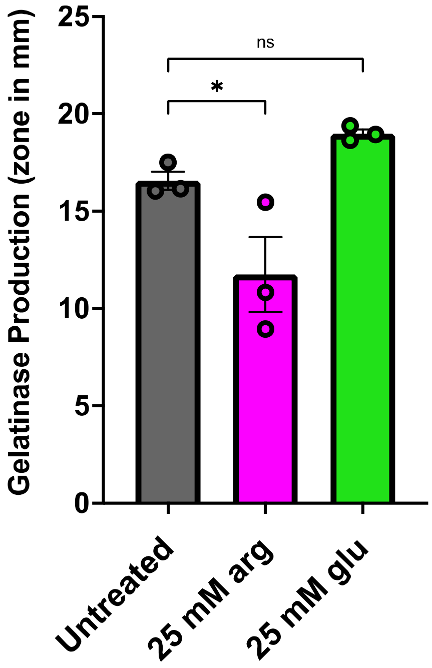

Supplementary Figure 2. Gelatinase activity of *E. faecalis* OG1RF grown with or without arginine. *E. faecalis* OG1RF cultures were spotted onto agar plates supplemented with 3% gelatin and 25 mM arginine or glucose. Hazy zones indicative of gelatinase activity were measured in mm after 24 hr growth. Each data point represents an individual biological replicate. Statistical significance was determined by one-way ANOVA with Dunnett's test for multiple comparisons. *p<0.05.

bioRxiv preprint doi: https://doi.org/10.1101/2024.05.30.596650; this version posted May 31, 2024. The copyright holder for this preprint (which was not certified by peer review) is the author/funder, who has granted bioRxiv a license to display the preprint in perpetuity. It is made available under aCC-BY-NC-ND 4.0 International license.

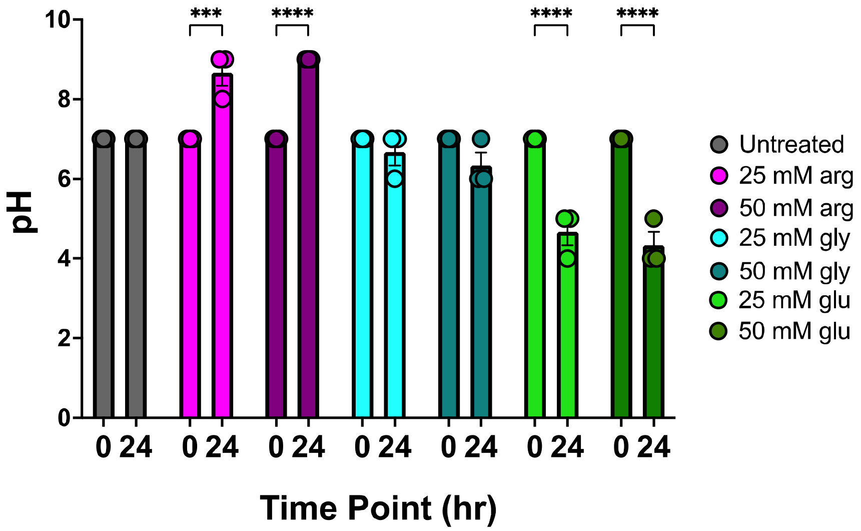

Supplementary Figure 3. pH of *E. faecalis* cultures. *E. faecalis* OG1RF was cultured in CBM supplemented with the indicated nutrients. pH measurements were taken at 0 hr prior to growth and after 24 hr incubation. Each data point represents an independent biological replicate. Error bars represent standard deviation. Statistical significance between 0 and 24 hr for each condition was assessed with two-way ANOVA and Sidak's test for multiple comparisons. ***p<0.001, ****p<0.0001
